# Transcriptome and metabolome analysis reveals mechanism of light intensity modulating iridoid biosynthesis in *Gentiana macrophylla* Pall.

**DOI:** 10.1186/s12870-024-05217-y

**Published:** 2024-06-11

**Authors:** Huanhuan Fu, Yaomin Wang, Fakai Mi, Li Wang, Ye Yang, Fang Wang, Zhenggang Yue, Yihan He

**Affiliations:** 1 State Key Laboratory of Research & Development of Characteristic Qin Medicine Resources (Cultivation), Shaanxi Innovative Drug Research Center, Co-construction Collaborative Innovation Center for Chinese Medicinal Resources Industrialization by Shaanxi & Education Ministry, School of Pharmacy, Shaanxi University of Chinese Medicine, Xianyang, 712046 P.R. China; 2 College of Life Science, Qinghai Normal University, Xining, 810016 P.R. China

**Keywords:** *Gentiana macrophylla* Pall., Transcriptome analysis, Metabolome analysis, Light intensity, Iridoid biosynthesis

## Abstract

Light intensity is a key factor affecting the synthesis of secondary metabolites in plants. However, the response mechanisms of metabolites and genes in *Gentiana macrophylla* under different light intensities have not been determined. In the present study, *G. macrophylla* seedlings were treated with LED light intensities of 15 µmol/m^2^/s (low light, LL), 90 µmol/m^2^/s (medium light, ML), and 200 µmol/m^2^/s (high light, HL), and leaves were collected on the 5th day for further investigation. A total of 2162 metabolites were detected, in which, the most abundant metabolites were identified as flavonoids, carbohydrates, terpenoids and amino acids. A total of 3313 and 613 differentially expressed genes (DEGs) were identified in the LL and HL groups compared with the ML group, respectively, mainly enriched in KEGG pathways such as carotenoid biosynthesis, carbon metabolism, glycolysis/gluconeogenesis, amino acids biosynthesis, plant MAPK pathway and plant hormone signaling. Besides, the transcription factors of *GmMYB5* and *GmbHLH20* were determined to be significantly correlated with loganic acid biosynthesis; the expression of photosystem-related enzyme genes was altered under different light intensities, regulating the expression of enzyme genes involved in the carotenoid, chlorophyll, glycolysis and amino acids pathway, then affecting their metabolic biosynthesis. As a result, low light inhibited photosynthesis, delayed glycolysis, thus, increased certain amino acids and decreased loganic acid production, while high light got an opposite trend. Our research contributed significantly to understand the molecular mechanism of light intensity in controlling metabolic accumulation in *G. macrophylla*.

**Supplementary Information:**

The online version contains supplementary materials available at 10.1186/s12870-024-05217-y.

## Introduction

*Gentiana macrophylla* Pall. (Gentianaceae) is an important herbal medicine with a variety of pharmacological activities [1]. Its major secondary metabolites are iridoids, the main components as loganic acid, swertiamarin, gentiopicroside and sweroside [2], showing multiple pharmacological effects such as antidiabetic [3, 4], anti-proliferative [5], hepatoprotective, anti-inflammatory [6], antioxidant [7] and neuroprotective [8]. The resource of *G. macrophylla* is widely distributed in almost all regions of northern China, across 33°N ~ 53°N to 103°E ~ 135°E, and altitudes ranging from 400 to 2400 m. Due to the complex distribution of the natural environment, the quality of herbal materials is extremely variable. Several researches have observed ecological factors could influence the contents of the secondary metabolites in *G. macrophylla* [9, 10]. Right now, the underlying mechanism could be further explored, owing to the published data of *G. macrophylla* genome in 2022 [11] and the progression of iridoid biosynthetic pathway revealed in different plants [12, 13].

Light, as one of the most important ecological factors, has been observed to affect plant growth and development, and the production of secondary metabolites in medicinal plants can be regulated under different light-emitting diodes (LEDs) or other light sources [14]. Three light environment factors: light intensity, light quality and photoperiod [15] were always investigated during the research procedure. For example, exposure to different light qualities, the content of artemisinin in *Artemisia annua* shoots was enhanced by 2.3, 2.5 and 1.9 folds under white, blue and red light, respectively [16]; the accumulation of coumaeoylquinic acid in *Hypericum perforatum* leaves was increased by 89%, 65%, 84% and 74% under white, blue, red and green light, respectively [16]. Treated with different light intensities, the content of total sesquiterpenoids was highest in the rhizome of *Atractylodes lancea* under 80% mild shading compared to 100% strong and 7% low light [17]; the contents of total phenols and flavonoids in *Polygonum minus* were significantly increased under 50% and 70% shade, respectively [18]; the content of saponarin in Barley Sprouts was decreased along with the reduction of light intensity [19]; the synthesis of monoterpene in *Lilium* ‘Siberia’ was increased under light intensities of 200 and 600 µmol/m^2^/s [20]; 23 terpenoid derivatives were identified in the leaves of *Pinus massoniana*, while, most of which were decreased under low light treatment [21].

The mechanism of photoregulation was also investigated. Higher altitude with strong light intensity increased the expression of genes involved in light stress (*HSP18.1*, *HSP70*, *UBC4*, *ERF5*, *ERF9*, *APX3* and *EX2*) and genes involved in flavonoid biosynthesis (*PAL*, *CHS1*, *IFRL*, *ANS*, *MYB4*, *bHLH137*, *CYP6*, *PPO1* and *ABCB19*), thus promoting the accumulation of flavonoids, flavonols, and anthocyanins in *Sinopodophyllum hexandrum* [22]. Three key genes in the anthocyanin biosynthesis pathway (*CHI*, *DFR*, and *ANS*) and 147 transcription factors (MYB, bHLH, bZIP, ERF, and NAC) involved in malonylshisonin biosynthesis were identified [15]. In *Angelica dahurica*, under 90% natural light, coumarin biosynthesis could be decreased by reducing the expression of *COMT* and *4CL* genes [23]. In *Ginkgo biloba*, under 100% sunlight, levels of flavonols (total flavonol, quercetin, kaempferol, and isorhamnetin) in leaves were increased by enhancing the expression of flavonoid biosynthesis-related genes (*PAL*, *CHS*, *F3H*, and *FLS*) [24]. In *Lonicera macranthoides* leaves, reduction of light intensity decreased the expression of *CHS* in leaves, then reduced the accumulation of chlorogenic acid [25]. In *Lonicera japonica*, high light intensity enhanced the production of luteoloside and total flavonoids in flower buds, by increasing the expression of genes involved in flavonoid biosynthesis (*PAL*, *4CL*, *C4H*, *CHS*, and *CHI*) [26]. Thus, these results indicated that light might act directly on photoregulatory genes, and subsequently affect critical genes in secondary metabolite biosynthesis, thereby activate or inhibit metabolites accumulation in plant tissues.

In our study, the mechanism of iridoid metabolites as the main active ingredients in *G. macrophylla* leaves in response to light intensity was investigated. The *G. macrophylla* seedlings were treated with different light intensities, and then variation analysis of metabolite levels and their regulator gene levels were detected via metabolome and transcriptome; correlation analysis between iridoids and differentially expressed transcription factors (TFs) and correlation analysis between differentially expressed TFs and differentially expressed genes (DEGs) involved in iridoid biosynthesis were performed. The results showed that light intensity could modulate iridoid biosynthesis in *G. macrophylla*, and the underlying mechanism was revealed as different light intensities altering the photosynthetic rate, sequentially regulating the primary metabolic system, the secondary metabolic pathway, the key responsive TFs and the light-related hormone signaling, thereby increasing or decreasing the accumulation of iridoids. This study provides new insights into the molecular mechanism underlying the biosynthesis and regulation of iridoids in *G. macrophylla*.

## Materials and methods

### Plant materials and treatments

*G. macrophylla* seedlings were grown in the laboratory at 26 ± 1 °C and 68% humidity for 12 h per day. At the six-leaf stage (grown for 6 months), seedlings were exposed to different light intensities: the photosynthetic photon flux density (PPFD) of the broad spectrum LED lights was set at 15 µmol/m^2^/s (low light, LL), 90 µmol/m^2^/s (medium light, ML), or 200 µmol/m^2^/s (high light, HL), with a photoperiod of 14 h light and 10 h dark (day/night conditions). Each treatment group was treated with three pots and each pot contained 30 plants. Three biological replicates were performed for each treatment group. Five-day-old samples were collected for further analysis. Leaves of the three treatment groups were collected and then divided into three parts: one part was immediately frozen in liquid nitrogen and stored at -80 °C for RNA extraction, metabolomic and transcriptomic analysis; one part was used to determine the contents of Chls and carotenoid; and the remaining part was dried to determine the contents of iridoids.

### Determination of the contents of Chls and carotenoid

For each sample, 0.1 g of fresh leaf sample was extracted in 10 mL of 95% ethanol, then 200 µL extraction solution was added to a 96-well plate and the absorbance values (A) were measured at wavelengths of 665 nm, 649 nm and 470 nm in a microplate reader. Each group was replicated three times. The content of Chl was calculated as Chl = Chl a + Chl b, Chl a = (13.95 × A_665_ − 6.88 × A_649_) V/1000 × W, and Chl b = (24.69 × A_649_ − 7.32 × A_665_) V/1000 × W. The content of carotenoid was calculated as carotenoid = (1000 × A_470_ − 2.05 × Ca − 114.8 × Cb)/(245 × 1000 × W) [27]. The results were expressed in mg/g, where Ca was the concentration of Chl a, Cb was the concentration of Chl b, V was the volume of the extract, and W was the wight of the fresh leaf.

### Determination of the contents of iridoid metabolites

For each sample, 20 mg of dry weight sample was extracted in 1 mL methanol by ultrasonication for three times for 40 min each; centrifuged for 5 min at 12,000 rpm, and the liquid supernatant was combined and filtered through a 0.22 μm membrane. The levels of four major iridoid metabolites, loganic acid, sweroside, swertiamarin and gentiopicroside, were determined by LC-20ADXR HPLC (Shimadzu, Japan) (Welch 4.6 mm × 250 mm, C18 5 μm particles (Welch, China); UV detector, 254 nm; column temperature, 30 °C; flow rate, 0.8 mL/min), using the constant solvent system ACN (Welch, China)/H_2_O (with 0.1% phosphoric acid) as the mobile 0–35 min, ACN 10%. Each injection volume was set to 10 µL.

The standard curve was constructed from the metabolite content (*X*, mg/mL) and peak area (*Y*). The linear regression equation was *Y* = 1,602,377.2743 *X* + 7,867.6622 (linear range 0.00214814 mg/mL – 1.1 mg/mL, *R*^2^ = 0.9996) for loganin acid (Herbest, China), *Y* = 2,933,123.5429 *X* + 108.4713 (linear range 0.0158 mg/mL – 1.0112 mg/mL, *R*^2^ = 1.0000) for swertiamarin (Push, China), *Y* = 3,930,675.6374 *X* − 5,906.8466 (linear range 0.03225 mg/mL – 4.128 mg/mL, *R*^2^ = 0.9999) for gentiopicroside (Yuanye, China), *Y* = 18,813,950.6495 *X* + 12,425.4530 (linear range 0.0009875 mg/mL – 1.0112 mg/mL, *R*^2^ = 1.0000) for sweroside (Push, China). Each group was replicated three times.

### Transcriptome analysis of DEGs

The transcriptome sequencing was performed by the Beijing Genomics Institute (BGI) (Shenzhen, China) (https://www.genomics.cn/). The *G. macrophylla* reference genome was obtained from the following link (https://www.ncbi.nlm.nih.gov/ass-embly/GCA_026214975.1) [11]. The HISAT2 software (version 2.0.1) [28] was used for alignment to map the filtered reads to the reference genome. Differential analysis of three groups was performed using DESeq2 [29] to identify DEGs. The false discovery rate (*FDR*) correction *p*-value was calculated using the Benjamini-Hochberg method, and DEGs were identified according to *FDR* ≤ 0.05 and |log2 fold change| ≥ 1.0 [30].

The National Center for Biotechnology Information (NCBI), Non-redundant (Nr, http://ftp.ncbi.nih.gov/blast/db/FASTA/nr.gz), Kyoto Encyclopedia of Genes and Genomes (KEGG, https://www.kegg.jp/), and Gene Ontology (GO, http://geneontology.org/) databases were used for gene function annotation. DEGs were functionally classified by GO and KEGG annotation, the phyper function of the *R* software was used for enrichment analysis, and *FDR* ≤ 0.05 was considered as significant enrichment. The correlations of DEGs, differentially expressed TFs and iridoids were analyzed and heatmaps were drawn using metware cloud (https://cloud.metware.cn/#/home), and the network diagram was drawn using Cytoscape.

### LC-MS analysis of metabolite profiles

Metabolite profiling was conducted using an untargeted metabolome method at BGI (Shenzhen, China) (https://www.genomics.cn/). For each sample, 100 mg of the fresh samples was weighted into 1.5 mL Eppendorf tubes and soaked in 800 µL of pre-cooled extraction solution (MeOH : H_2_O = 7 : 3, v/v) together with 20 µL of internal standard (d_3_-Leucine, ^13^C_9_-Phenylalanine, d_5_-Tryptophan, ^13^C_3_-Progesterone). The mixture was homogenized using a weaving grinder at 50 Hz for 300 s, followed by ultrasonication in a water bath at 4 °C for 10 min. After standing for 2 h at -20 °C, the extracts were centrifuged at 25000 rcf at 4 °C for 15 min. 600 µL of the supernatant was filtered through a 0.22 μm membrane. Additionally, 20 µL of filtered solution from each sample was combined to create a mixed QC sample, which was used to access the repeatability and stability of the LC/MS analysis.

In this experiment, Waters 2777 C UPLC (Waters, USA) was used in series with Q exactive HF high resolution mass spectrometer (Thermo Fisher Scientific, USA) for the separation and detection of metabolites. Chromatographic conditions: chromatographic separation was performed on BEH C18 column (1.7 μm, 2.1 × 100 mm, Waters, USA), with mobile phase A consisting of 0.1% formic acid in water and mobile phase B consisting of 0.1% formic acid in methanol under positive ion detection mode, and mobile phase A consisting of 10 mM ammonium formate in water and mobile phase B consisting of 10 mM ammonium formate in 95% methanol under negative ion detection mode. The column temperature was maintained at 45 °C. The gradient solvent system was as follows: 2% B over 0.0–1.0 min; 2%–98% B over 1.0–9.0 min; 98% B over 9.0–12.0 min; 98% B –2% B over 12.0–12.1 min; 2% B over 12.1–15.0 min. The flow rate was 0.35 mL/min and the injection volume was 5 µL. Mass spectrometry conditions: primary and secondary mass spectrometry data acquisition was performed using Q Exactive HF (Thermo Fisher Scientific, USA).

After importing the off-line data of mass spectrometry data into Compound Discoverer 3.3 software (Thermo Fisher Scientific, USA) and analyzing the mass spectrometry data in combination with BMDB (BGI metabolome database), and *m/z* cloud database and chemspider online database, a data matrix containing information such as metabolite peak area and identification results would be obtained. Then data preprocessing was performed using metaX [31] to obtain all forms of compounds and their corresponding intensities for subsequent data analysis. Differentially accumulated metabolites (DAMs) were identified using thresholds of variable significance of *p* -value ≤ 0.05 and fold change ≥ 2 or ≤ 0.5. Classification and functional annotation analysis was performed to obtain KEGG ID, category, and KEGG pathway of the identified metabolites, compared with KEGG and HMDB databases.

### Genes expression analysis by RT-qPCR

Total RNA was isolated using the Polysaccharide Polyphenol Plant Total RNA Extraction Kit (Bioteke, Beijing). The concentration and purity of RNA were detected by Nanodrop (Thermo Fisher, USA) and RNA integrity was detected by 1% agarose gel electrophoresis (Solarbio, Beijing), followed by cDNA synthesis using the PrimeScript™ IV 1st Strand cDNA Synthesis Mix (TaKaRa, Dalian), and stored at -20 °C. RT-qPCR was performed with the qTOWER 2.0 system (Analytik Jena, Germany) under the following cycling conditions: 95 °C for 30 s; 95 °C for 5 s and 60 °C for 20 s, 40 cycles. The relative expression of the genes was calculated by the 2^−∆∆Ct^ method. The *SAND1* gene was used as an internal reference [32]. Each group was replicated three times. The NCBI website (https://www.ncbi.nlm.nih.gov/tools/primerblast/index.cgi?LI-NK_LOC=BlastHome) was used to design the primers for the target genes (Additional file 1: Table S1).

### Statistical analysis

Statistical analysis of the data was performed using Excel, and the results were presented as means ± *SD*. Graph Pad Prism 8.0 software was used for the contents of Chls, carotenoid and four major iridoids, and different treatment groups were analyzed using *t*-test (*p* ≤ 0.05). IBM SPSS Statistics 26 software was used to analyze the metabolomic data, and different treatment groups were analyzed using one-way ANOVA with LSD multiple comparison tests (*p* ≤ 0.05).

## Results

**Fig. 1:**
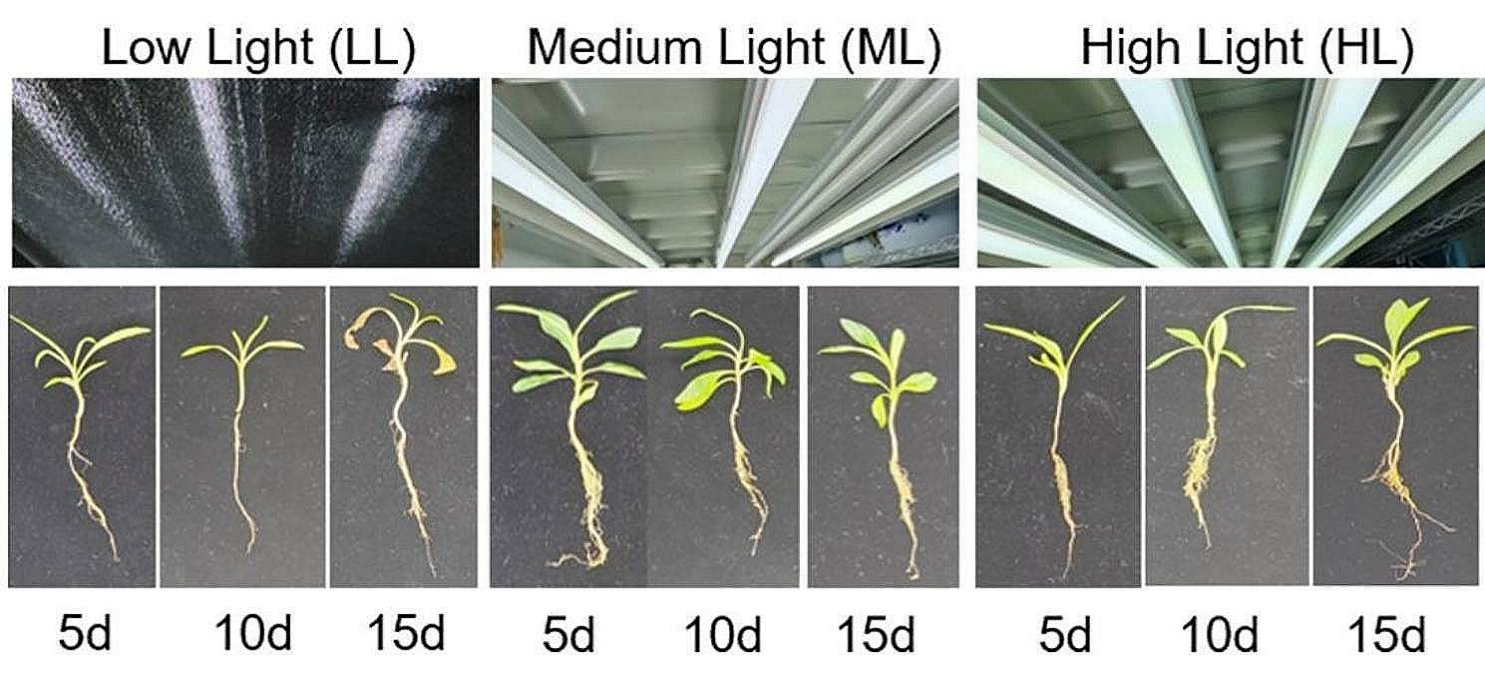
The growth conditions of *G. macrophylla* seedlings under different light intensity treatments on the 5th, 10th and 15th days, respectively. LL, ML and HL indicates the low, medium and high light treatment group, respectively

Compared with HL and ML groups, the plant morphology of seedlings under low light intensity treatment, showed different growth trends: the seedlings appeared clear phototaxis for the apical leaves were presented apparent vertical growth tendency on the 5th day, while, all leaves turned yellow and gradually withered as the time extended to the 15th day (Fig. 1). Considering the status of the leaf growth and the plant integrity, the 5-day-old samples were collected for further analysis.

### The variation of Chls and carotenoid contents at different light intensities

**Fig. 2:**
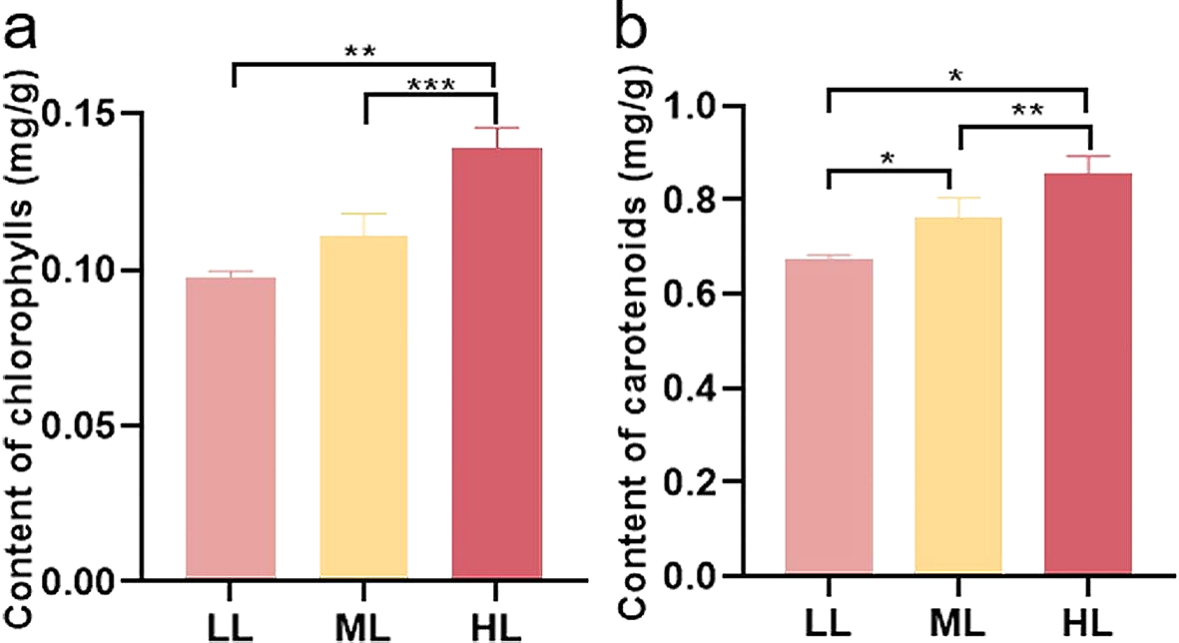
The contents of chlorophylls and carotenoid under different light intensity treatments at 5-day in *G. macrophylla* seedlings leaves. (**a**) Chlorophylls content. (**b**) Carotenoid content. *Statistically significant differences (* *P* < 0.05, ** *P* < 0.01, and *** *P* < 0.001)

Chls and carotenoid are two kinds of products produced during photosynthesis, and their contents can represent the levels of photosynthesis. As shown in Fig. 2, the contents of Chl and carotenoid were gradually increased along with the increase of light intensity, suggesting that our model was appropriate for studying the influence of different light intensities on the growth and development of *G. macrophylla*.

### The regulation of different light intensities on the levels of iridoid metabolites

**Fig. 3:**
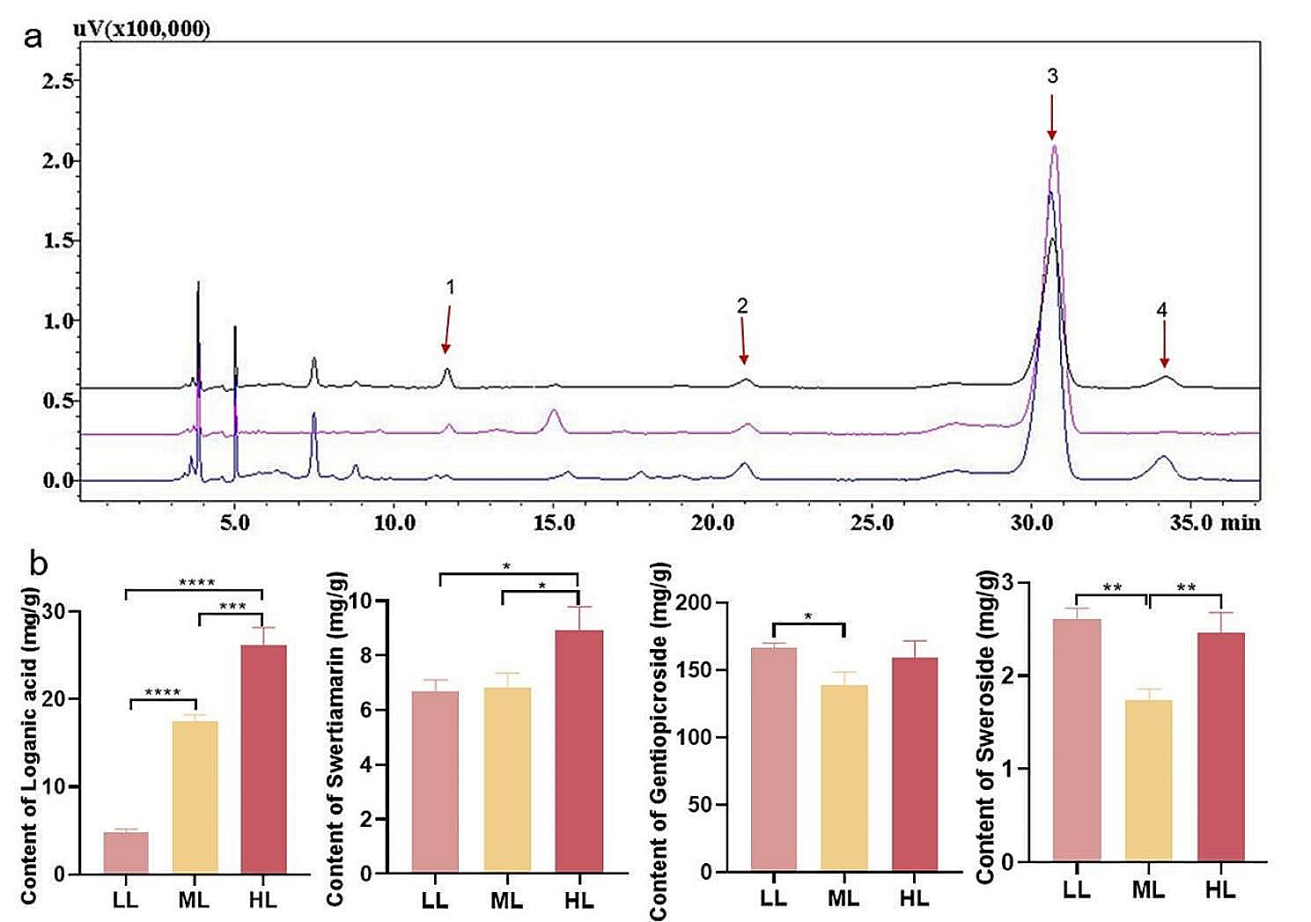
HPLC content determination of four major iridoid metabolites at 5-day. (**a**) Black, pink, and blue represents chromatogram of ML, LL, and HL, respectively, while, 1, 2, 3 and 4 represents chromatographic peaks of loganic acid, swertiamarin, gentiopicroside and sweroside, respectively. (**b**) Determination of levels of four major iridoid metabolites. *Statistically significant differences (* *P* < 0.05, ** *P* < 0.01, *** *P* < 0.001, and **** *P* < 0.0001)

As loganic acid, swertiamarin, gentiopicroside and sweroside were four major active components, their contents could represent the degree of metabolites accumulation during the growth and development of *G. macrophylla* (Fig. 3). Compared HL to ML group, all four components exhibited a significant increasing trend, and the contents of loganic acid, swertiamarin, gentiopicroside and sweroside increased by 49.44%, 30.54%, 14.67% and 40.94%, respectively. Compared LL to ML group, the accumulation of loganic acid and swertiamarin showed opposite trends with gentiopicroside and sweroside. The levels of loganic acid and swertiamarin decreased by 72.39% and 2.21%, while, the levels of gentiopicroside and sweroside increased by 19.77% and 49.62%, respectively (Fig. 3). These results suggested that under low light intensity treatment, self-protection function was triggered in plants, thus some metabolites crucial for plant survival, such as gentiopicroside in *G. macrophylla*, might be produced in respond to the external stimulus.

### Metabolomic analysis of metabolites at different light intensities

**Fig. 4:**
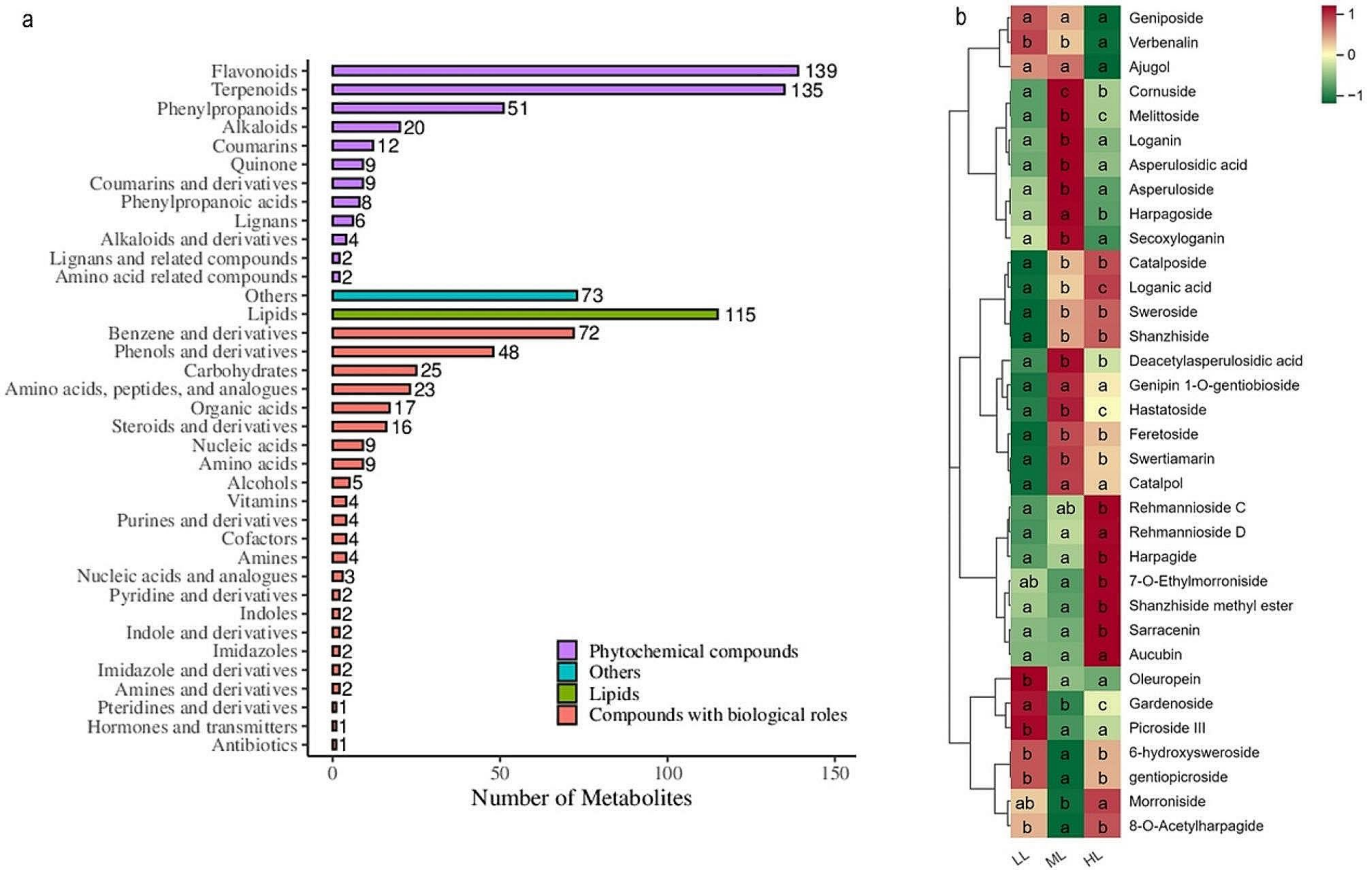
Untargeted metabolome analysis of the metabolic composition in *G. macrophylla* seedling leaves treated with different light intensities at 5-day. (**a**) Bar diagram depicts metabolites classification. The x-axis and y-axis represents the numbers and the classes of metabolites, respectively. (**b**) Hierarchical clustering heatmap analysis of iridoid compounds by *z*-score. The color represents the scale of iridoids contents. Red indicates upregulation, while, green indicates downregulation. Lowercase letters represent a significant difference of *p* ≤ 0.05 in the same iridoid metabolite among the treatments

As shown in Fig. 4a, a total of 2162 metabolites were identified by the untargeted metabolome analysis and categorized into 38 classes according to their structural features, including flavonoids (139, comprising 6.43%), terpenoids (135, comprising 6.24%), lipids (115, comprising 5.32%), benzene and derivatives (72, comprising 3.33%), phenylpropanoids (51, comprising 2.36%), phenols and derivatives (48, comprising 2.22%), carbohydrates (24, comprising 1.11%), amino acids, peptides, and analogues (23, comprising 1.06%), alkaloids (20, comprising 0.93%), organic acids (17, comprising 0.79%), other compound types (198, comprising 9.16%), and unknown classification (1320, comprising 61.05%). Among them, 32 iridoid metabolites were identified, including 7 secoiridoids as gentiopicroside, swertiamarin, sweroside, loganic acid, loganin, secoxyloganin and 6-hydroxysweroside (Fig. 4b). Moreover, the variations in the levels of loganic acid and gentiopicroside were largely consistent with the HPLC determined results (Fig. 3b).

**Fig. 5:**
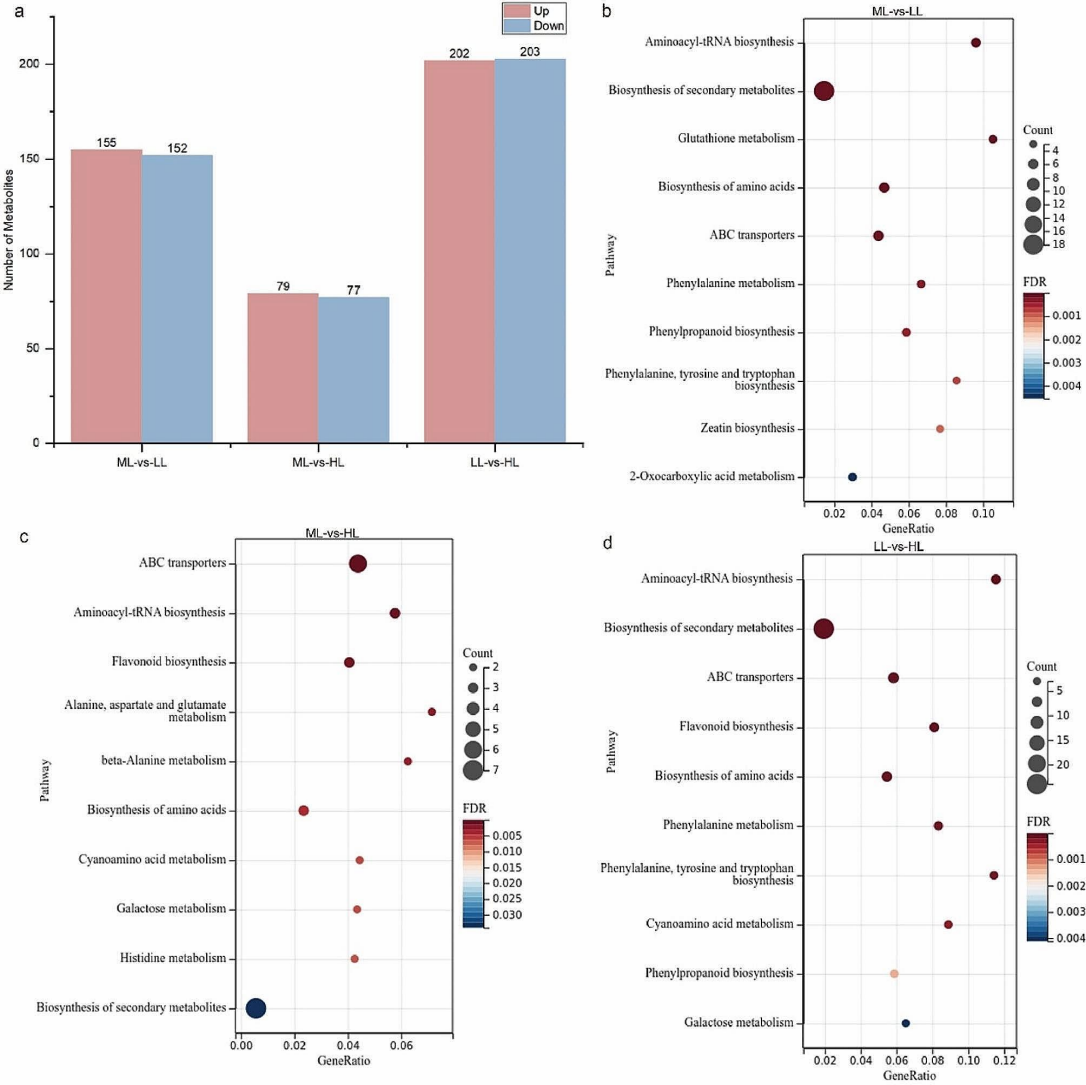
Untargeted metabolome analysis of differentially accumulated metabolites (DAMs) in the ML-vs.-LL, ML-vs.-HL, and LL-vs.-HL comparisons at 5-day. (**a**) Numbers of DAMs. Red indicates upregulation, while, blue indicates downregulation. (**b**-**d**) KEGG enrichment analysis of the DAMs

DAMs between pairs of samples (ML vs. LL group, ML vs. HL group, and LL vs. HL group) were determined on the basis of variable significance with a *p*-value ≤ 0.05 and a fold change ≤ 0.5 or ≥ 2. Thus, 307, 156 and 405 DAMs were distinguished in the ML vs. LL, ML vs. HL, and LL vs. HL group, respectively, with the number of upregulated DAMs to be 155, 79, and 202, and the number of downregulated DAMs to be 152, 77, and 203, respectively (Fig. 5a).

**Fig. 6:**
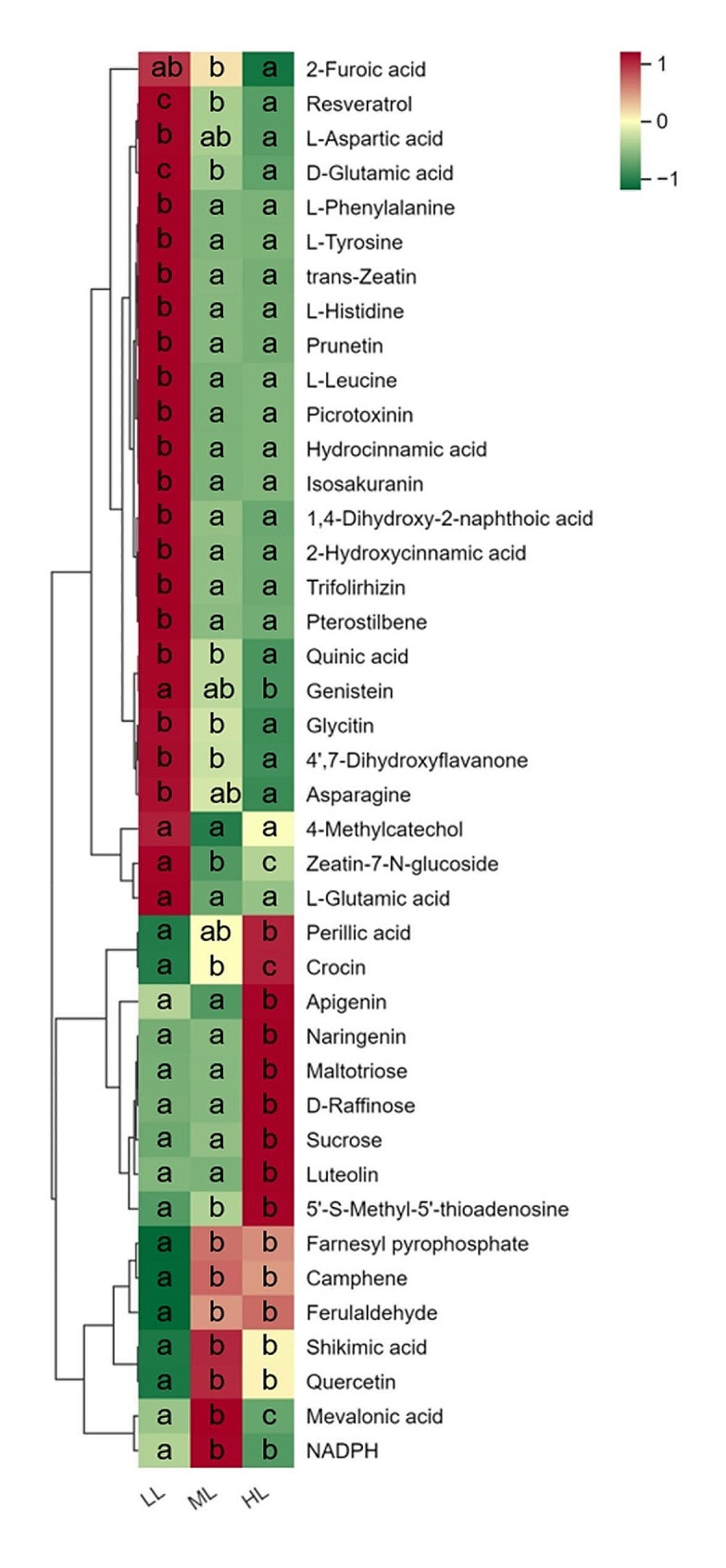
The contents of the 41 identified DAMs refer to KEGG pathway. The color represents the scale of metabolites contents. Red indicates upregulation, while, green indicates downregulation. Lowercase letters represent a significant difference of *p* ≤ 0.05

Among the total 544 DAMs, 41 compounds mainly as flavonoids, carbohydrates, terpenoids, and amino acid compounds (Fig. 6), were enriched in KEGG pathways such as metabolic pathway, aminoacyl-tRNA biosynthesis, secondary metabolite biosynthesis, amino acids biosynthesis, and ABC transporters (Fig. 5b-d). In addition, after clustering analysis, they were roughly divided into two branches significantly responding to light intensity (Fig. 6, Additional file 2: Table S2). Thus, the results indicated that light intensity regulated metabolite biosynthesis in *G. macrophylla via* influencing the function of carbon metabolism, glycolysis/gluconeogenesis, plant hormone signal transduction and photosynthesis.

### Transcriptomic analysis of DEGs under different light intensities

A total of 9 group samples collected on the 5th day were performed transcriptome sequencing, and 24.32 GB of clean data (GC content ranging from 42.97% to 43.42%) were acquired within each sample exceeding 2.5 GB, in which, the Q30 base percentage for each library was ≥ 91.75% was considered as high quality reads for further analysis. In addition, more than 95% of the clean reads could be mapped to the reference genome (Additional file 3: Table S3).

### GO analysis

**Fig. 7:**
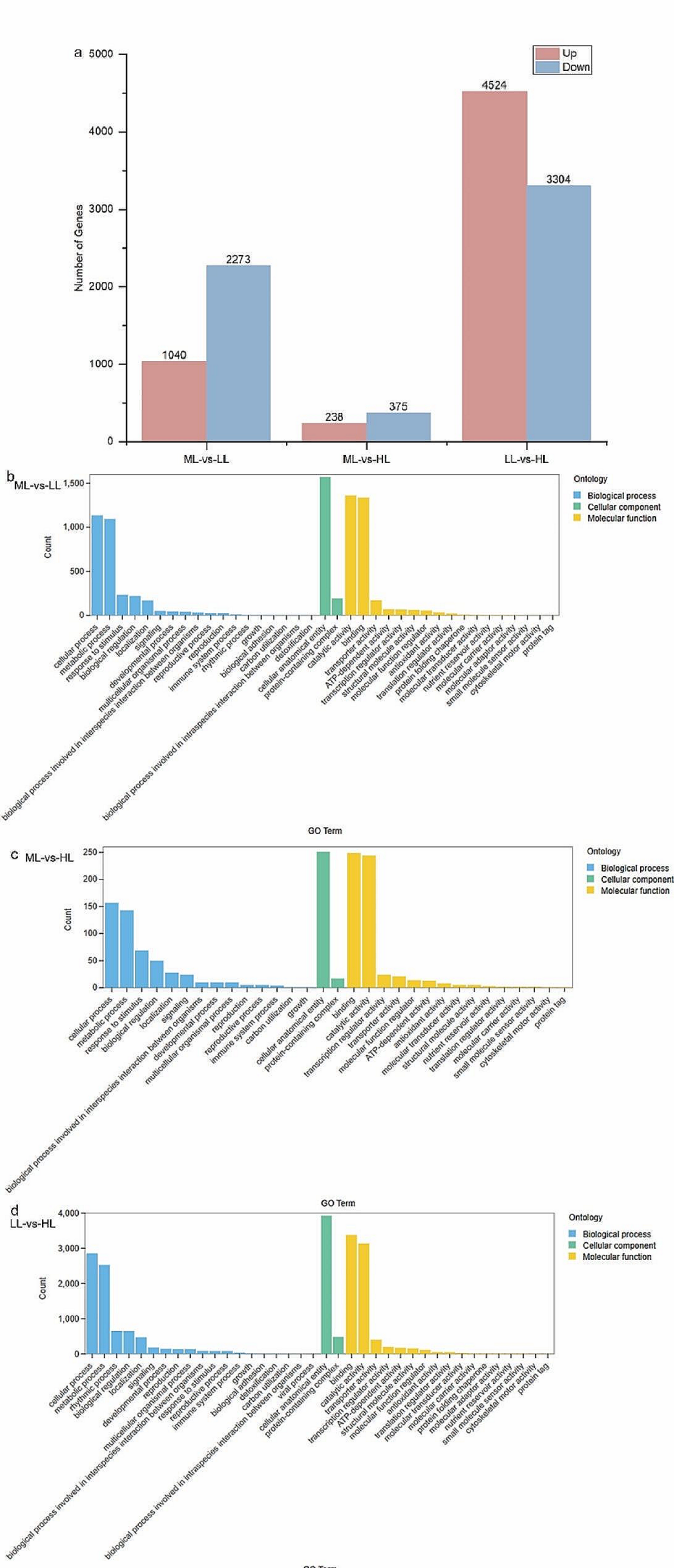
Analysis of differentially expressed genes (DEGs) in the ML-vs.-LL, ML-vs.-HL, and LL-vs.-HL comparisons at 5-day. (**a**) Numbers of DEGs. Red indicates upregulation, while, blue indicates downregulation. (**b**-**d**) Bar diagrams of GO classifications of DEGs

**Fig. 8:**
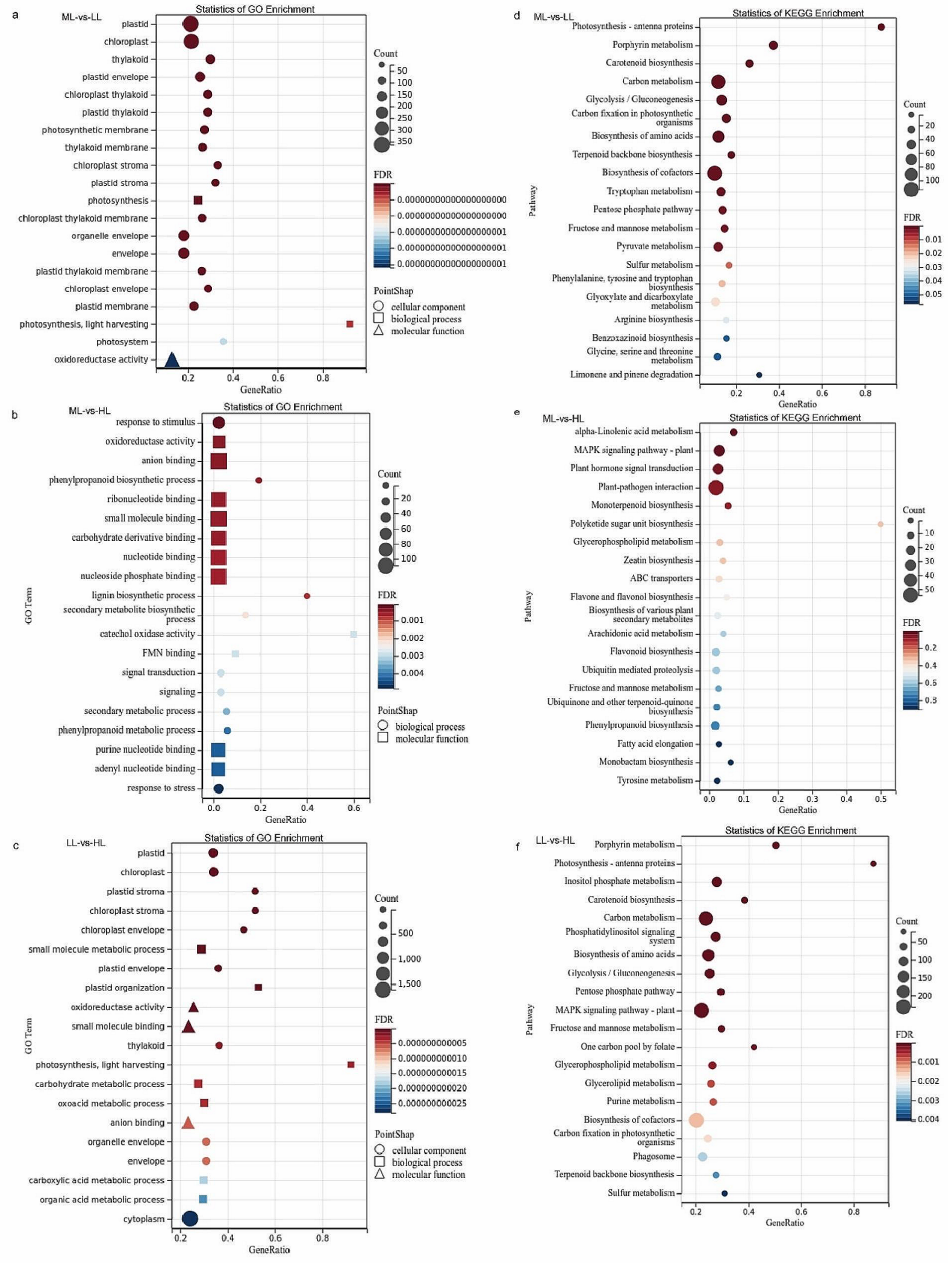
Enrichment analysis of DEGs in the ML-vs.-LL, ML-vs.-HL, and LL-vs.-HL comparisons. (**a**-**c**) GO enrichment analysis of the DEGs. (**d**-**f**) KEGG enrichment analysis of the DEGs

A total of 3313, 613, and 7758 numbers of DEGs were obtained in the ML vs. LL, ML vs. HL, and LL vs. HL group, respectively (Fig. 7a). After GO enrichment analysis was performed, only 2432, 436, and 5410 numbers of DEGs were enriched in the respective cluster, and primarily grouped into three main categories: biological processes, molecular functions, and cellular components. Furthermore, the three categories in each comparison group were further subdivided into 37, 31, and 38 smaller numbers of functional categories, respectively (Fig. 7b-d). The top 20 categories (the final 20 ones with the lowest *FDR* values) were selected for GO enrichment analysis (Fig. 8a-c).

GO enrichment analysis of ML vs. LL group revealed that DEGs were particularly enriched in organelles and cell membranes such as plastid, chloroplast, thylakoid, plastid membrane, and photosynthetic membrane of the cellular component category; DEGs were mainly associated with photosynthesis, including both photosynthesis and photosynthetic light harvesting of the biological process category; DEGs were notably enriched in enzyme activity and catalytic activity, encompassing oxidoreductase activity, carbohydrate phosphatase activity, catalytic activity and sugar phosphatase activity of the molecular function category (Fig. 8a).

GO enrichment analysis of ML vs. HL group revealed that DEGs were significantly enriched in metabolic processes, signal transductions, defense reactions such as response to stimulus, phenylpropanoid biosynthesis process, lignin biosynthesis process, secondary metabolite biosynthesis process, signal transduction, signaling and secondary metabolic process of the biological process category; DEGs were notably enriched in enzyme activities and binding, such as oxidoreductase activity, anion binding, ribonucleotide binding, small molecule binding, and carbohydrate derivative binding of the molecular function category (Fig. 8b).

GO enrichment analysis of LL vs. HL group revealed that DEGs were significantly enriched in organelles, cell membranes and cytoplasm such as plastid, chloroplast, thylakoid, envelope and the cytoplasm of the cellular component category; DEGs were mainly enriched in plastid organizations and metabolic processes, such as small molecule metabolic process, plastid organization, carbohydrate metabolic process, oxoacid metabolic process, carboxylic acid metabolic process, and organic acid metabolic process of the biological process category; DEGs were notably enriched in enzyme activities and bindings such as oxidoreductase activity, small molecule binding, and anion binding of the molecular function category (Fig. 8c).

### KEGG analysis

A total of 1619 numbers of annotated genes were associated with 132 numbers of KEGG pathways in the ML vs. LL group, in which 19 KEGG pathways were differentially represented (*FDR* ≤ 0.05) (Fig. 8d), such as carotenoid biosynthesis (ko00906), carbon metabolism (ko01200), glycolysis/gluconeogenesis (ko00010), carbon fixation in photosynthetic organisms (ko00710), biosynthesis of amino acids (ko01230), terpenoid backbone biosynthesis (ko00900), tryptophan metabolism (ko00380), biosynthesis of cofactors (ko01240), and pyruvate metabolism (ko00620).

A total of 321 numbers of annotated genes were associated with 101 numbers of KEGG pathways in ML vs. HL group, in which 4 KEGG pathways were differentially represented (Fig. 8e), such as alpha-linolenic acid metabolism (ko00592), MAPK signaling pathway-plant (ko04016), plant hormone signal transduction (ko04075), and plant-pathogen interaction (ko04626).

A total of 3955 annotated genes were associated with 137 KEGG pathways in the LL vs. HL comparison, in which 34 KEGG pathways were differentially represented (Fig. 8f), such as photosynthesis-antenna proteins (ko00196), carotenoid biosynthesis (ko00906), carbon metabolism (ko01200), biosynthesis of amino acids (ko01230), glycolysis/gluconeogenesis (ko00010), pentose phosphate pathway (ko00030), glycerophospholipid metabolism (ko00564), glycerolipid metabolism (ko00561), biosynthesis of cofactors (ko01240), carbon fixation in photosynthetic organisms (ko00710), and terpenoid backbone biosynthesis (ko00900).

### Identification of DEGs related to the iridoid biosynthesis pathway

**Fig. 9:**
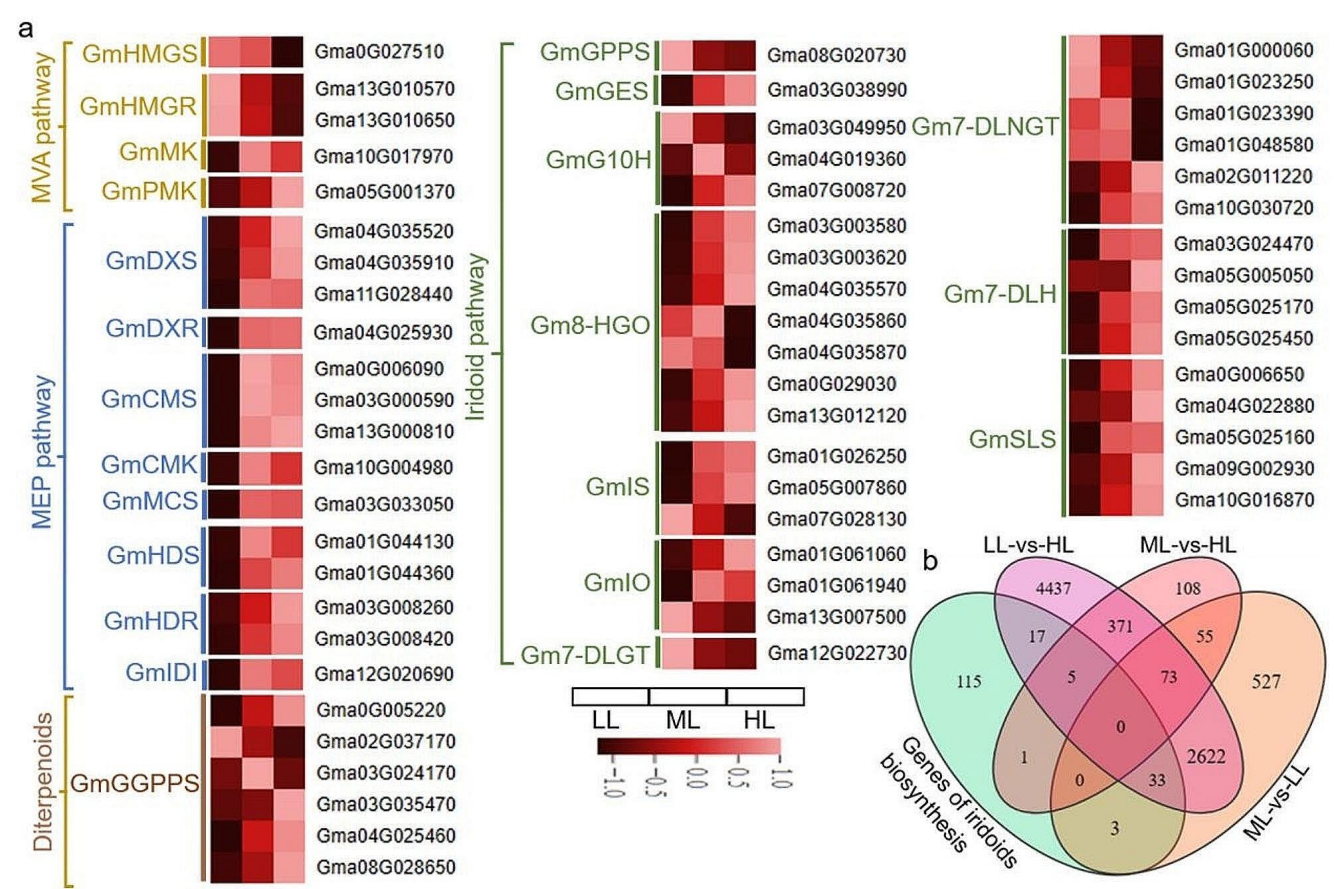
The expression of DEGs involved in iridoid biosynthesis at 5-day. (**a**) Heatmap of DEGs expression levels (normalized via the *z*-score). The color shading represents the expression levels. (**b**) Venn diagram depicting comparison of the DEGs numbers in the iridoid biosynthesis pathway

As mentioned above, iridoids were the major active components in *G. macrophylla*, and the genes related to the iridoid biosynthesis pathway in DEGs were chosen for further analysis. A total of 38, 6, and 61 numbers of DEGs belonging to 23 gene families related to the iridoid biosynthesis pathway were identified in the ML vs. LL, ML vs. HL, and LL vs. HL group, respectively (Fig. 9b). These gene families were depicted as *GmDXS*, *GmDXR*, *GmCMS*, *GmCMK*, *GmMCS*, *GmHDR*, *GmHDS*, *GmIDI*, *GmHMGS*, *GmHMGR*, *GmMK*, *GmPMK*, *GmGPPS*, *GmGGPPS*, *GmGES*, *GmG10H*, *Gm8-HGO*, *GmIS*, *GmIO*, *Gm7-DLGT*, *Gm7-DLNGT*, *Gm7-DLH*, and *GmSLS* (Additional file 4: Table S4). To visualize the changes in pathway DEGs in *G. macrophylla* leaves under different light intensity treatments, heatmaps were built (Fig. 9a). The results showed that under low light condition, the expression of most genes in the iridoid biosynthesis pathway was downregulated, including the upstream genes of *GmHMGS*, *GmHMGR*, *GmMK* and *GmPMK* in the MVA pathway and *GmDXS*, *GmDXR*, *GmCMS*, *GmCMK*, *GmMCS*, *GmHDS*, *GmHDR*, *GmIDI* in the MEP pathway, and the downstream enzyme genes of *GmGPPS*, *GmGES*, *GmG10H*, *Gm8-HGO, GmIS*, *GmIO*, *Gm7-DLNGT*, *Gm7-DLH* and *GmSLS* in the biosynthesis of secoiridiod backbone. On the contrary, the expression of most genes was upregulated after exposure to high light intensity. Accordingly, two inferences were obtained, one was that light intensity affected the expression on both of the upstream and downstream genes related to iridoid biosynthesis; the other was that the MEP pathway was the primary pathway with a greater number of DEGs compared to the MVA pathway.

**Fig. 10:**
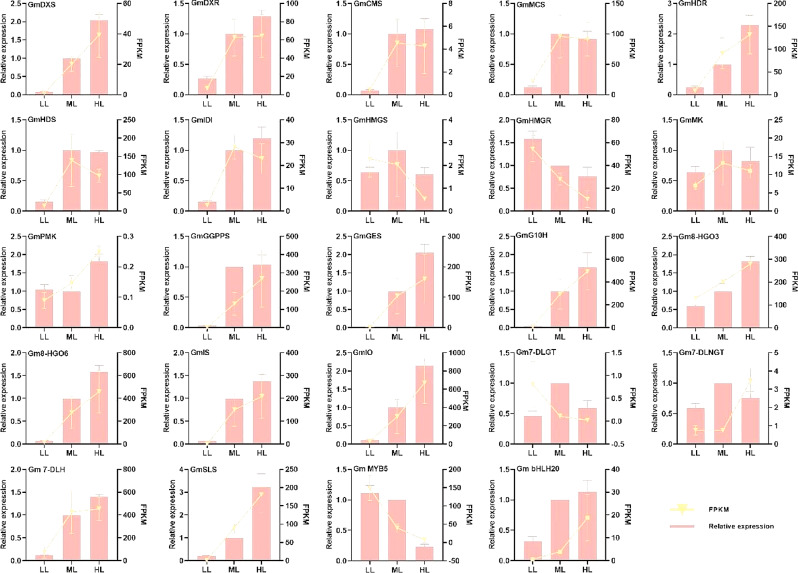
Determination of selected DEGs expression via RNA-seq and RT-qPCR comparison. The x-axis, left y-axis and right y-axis indicates the three treatment groups, RT-qPCR expression levels and the RNA-seq data with FPKM, respectively. Error bars indicate the standard deviation of three independent replicates

RT-qPCR was performed to verify the above DEGs in the iridoid pathway including 22 key genes of *GmDXS*, *GmDXR*, *GmCMS*, *GmMCS*, *GmHDR*, *GmHDS*, *GmHMGS*, *GmHMGR*, *GmMK*, *GmPMK*, *GmIDI*, *GmGGPPS*, *GmGES*, *GmG10H*, *Gm8-HGO3*, *Gm8-HGO6*, *GmIS*, *GmIO*, *Gm7-DLGT*, *Gm7-DLNGT*, *Gm7-DLH* and *GmSLS*. The transcript profiles of all selected genes were highly consistent with those obtained from the RNA-seq data (Fig. 10).

### Identification and analysis of TFs in response to light intensity

**Fig. 11:**
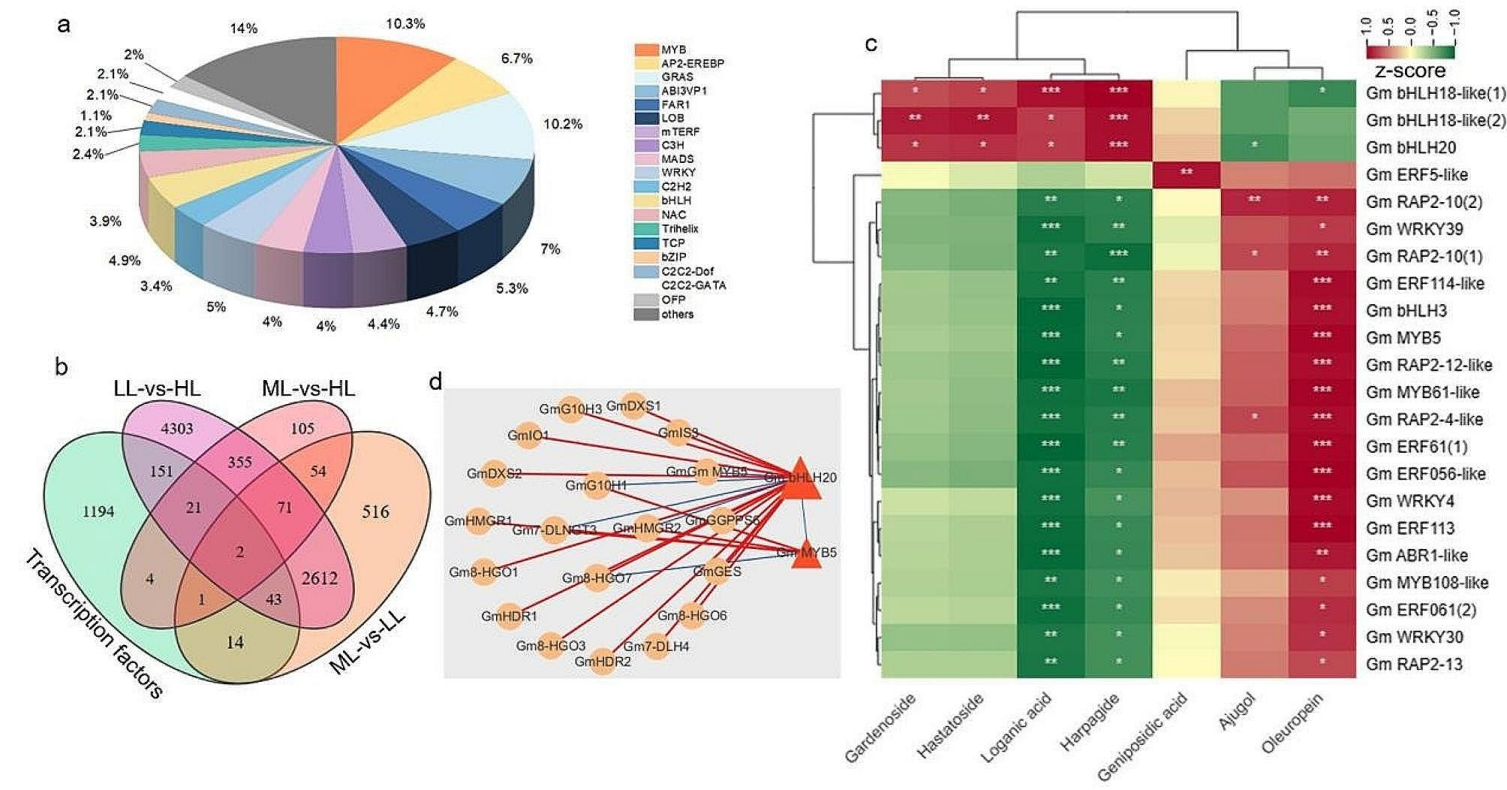
Analysis of transcription factors (TFs). (**a**) Distribution of TFs. (**b**) Venn diagram depicting comparisons of the differential TFs numbers. (**c**) Heatmap of correlations between differential TFs and iridoid compounds, *R* ≥ 0.8. (**d**) Correlation network diagram among the DEGs involved in iridoid biosynthesis, *GmbHLH20* and *GmMYB5*, *R* ≥ 0.8. Red lines indicate a positive correlation, while blue lines indicate a negative correlation. Line thickness indicates the strength of the correlation

A total of 1395 TFs in *G. macrophylla*, classified into 50 TF families, were identified using online data from PlantTFDB. Among these TF families, the most abundant were MYB (144 entries), AP2-EREBP (93 entries), bHLH (68 entries), NAC (55 entries) and WRKY (70 entries) (Fig. 11a). In particular, 2 TFs were differentially expressed in all three comparison groups, one entry was designated as *GmbHLH20* of the bHLH family, and the other entry was designated as *GmMYB5* of the MYB family (Fig. 11b).

RT-qPCR was performed to analyze *GmbHLH20* and *GmMYB5*, and the transcript profiles were highly consistent with RNA-seq data (Fig. 10).

### Correlation analysis of TFs, iridoids and genes related to iridoid biosynthesis

*GmbHLH20* was revealed to positively regulate the biosynthesis of loganic acid, while *GmMYB5* was negative ones, on the basis of correlation analysis (Pearson, *R* ≥ 0.8) between differentially expressed TFs and iridoid metabolites (Fig. 11c). Then, the correlation analysis of *GmbHLH20*, *GmMYB5* and the iridoid biosynthesis genes was performed (Pearson, *R* ≥ 0.8). The results showed that *GmbHLH20* was negatively correlated with *GmMYB5* (Fig. 11d); *GmbHLH20* might positively regulate the expression of *GmDXS1*, *GmDXS2*, *GmHDR1*, *GmHDR2*, *GmGES*, *GmG10H3*, *Gm8-HGO1*, *Gm8-HGO3*, *Gm8-HGO6*, *Gm8-HGO7*, *GmIS3*, *GmIO1*, *Gm7-DLH4* and *GmGGPPS6*, while negatively regulate the expression of *GmHMGR2*, *GmG10H1*and *Gm7-DLNGT3*; *GmMYB5* might positively regulate the expression of *GmHMGR1*, *GmHMGR2*, *GmG10H1* and *Gm7-DLNGT3*, while negatively regulate the expression of *Gm 8-HGO7*. Accordingly, it could be concluded that *GmbHLH20* and *GmMYB5* might play key roles in light intensity regulating iridoids biosynthesis.

## Discussion

Unlike animals, the plants are one of the species that cannot allodially move away from their native habitats, face different ecological factors, thus passively accept the environmental stimuli [33]. In order to adapt to the external environment, plants initiate internal regulation by producing metabolites to promote growth and development or to defend themselves [34]. Many studies have revealed that the accumulation of metabolites was influenced by the ecological factors [9, 10]. For *G. macrophylla*, intricate ecological conditions, such as various distribution, temperature, altitude, light and moisture, have been observed to influence the contents of iridoid metabolites [2, 10, 35], but the complex mechanisms have not been revealed yet. Thus, the herbal materials from different habitats are presented in different quality, which is unfavorable for clinical application.

Among the ecological factors, light is considered to be essential. LED light sources of different intensities have been found to have distinct effects on the plant growth [36, 37], the protective enzyme system [38], the photosynthesis [19], and the accumulation of secondary metabolites [19, 39, 40]. Photosynthesis plays a vital role in the oxidative respiratory chain as the primary driving force to initiate changes during plant growth and development. Within the photosynthetic system, accessory pigments such as various Chls, carotenoid, and phycobilins, serving as energy receptors, absorb the energy from photons and channel it to the primary reaction centers named as P680 in photosystem II and P700 in photosystem I for further photochemical processes [41]. For the contents of carotenoid and chlorophylls were increased or decreased along with the enhancement or reduction of the light intensity, their levels were used to evaluate the influence in photosynthetic system of light intensity [25, 42–44]. In this study, both Chls and carotenoid showed increased expression with increase of light intensity (15, 90 and 200 µmol/m^2^/s), as well as the expression of enzyme genes related to the synthesis of phytoene, lycopene, carotene, zeaxanthin, violaxanthin, and xanthoxin in the carotenoid biosynthesis pathway (map00906), and Chl a and Chl b in the porphyrin metabolism pathway (map00860) also displayed the same expression trend (Additional files 5–8: Figs. S1-S4, Additional file 9: Table S5 and Additional file 10: Table S6) in the KEGG differential gene enrichment analysis. Furthermore, with the prolongation of the light treatment duration, the leaves of *G. macrophylla* seedlings under low light gradually turned yellow and withered during the 15 days period, indicating a gradual loss of Chls leading to impaired photosystem. Thus, subsequent biochemical reactions would be triggered due to the alteration of photosynthesis.

The first consequence is that the impaired photosynthetic carbon assimilation would affect the primary metabolic pathways in plants, including amino acid metabolism, starch and sucrose metabolism [45]. When *G. macrophylla* was exposed to different light intensities, the levels of certain amino acids and their derivatives (L-glutamic acid, L-leucine, L-tyrosine, D-glutamic acid, L-phenylalanine, L-histidine, and asparagine) were presented higher accumulation in LL group compared to ML and HL groups, as well the expression of enzyme genes involved in the pathway reflected the same trend (Additional file 11: Fig. S5 and Additional file 12: Fig. S6, Additional file 13: Table S7). These findings were consistent with the recent research conclusion that the levels of several amino acids such as phenylalanine and tryptophan, were commonly upregulated in plants under diverse environmental conditions, such as hydropenia, cold stress and during dark-induced senescence [46]. However, the levels of maltotriose, d-raffinose, and sucrose were presented lower accumulation in LL group, as well as the expression of genes in the glycolysis, the pentose phosphate pathway, and the shikimate pathway (Additional file 12: Fig. S6). It is easy to understand that a decreased rate of photosynthetic activity brings out a reduction in the efficiency of converting light energy into chemical energy, thus the carbohydrate stores are depleted under low light intensity, but vital substances such as amino acids were primarily accumulated for survival [47] (Additional file 11: Fig. S5).

The second consequence is that the contents of certain carbohydrates decrease after exposure to low light, such as maltotriose, d-raffinose, and sucrose, while they increase under high light condition. For example, the levels of soluble sugar and starch significantly decreased after shade treatment of *Lonicera macranthoides* [25]. For the glycolysis/gluconeogenesis pathway is a metabolic process, involving in the conversion of 6-carbon glucose to 2-carbon molecules, then bonding with the tricarboxylic acid cycle [48]. It suggests that glycolysis/gluconeogenesis deferred by impaired photosynthetic carbon assimilation, would affect the secondary metabolites pathway in plants [19]. So far, the biosynthesis pathway of iridoids was partially revealed as initiating from MVA or MEP pathway to generate IPP and DMAPP, then, under the geranyl diphosphate synthase *GES*, geraniol 8-hydroxylase *G10H*, 8-hydroxygeraniol dehydrogenase *8-HGO*, (S)-8-oxocitronellyl enol synthase *IS*, 7-deoxyloganetic acid glucosyltransferase *7-DLGT*, and 7-deoxyloganate 7-hydroxylase *7-DLH* to successively generate geraniol, 8-hydroxygeraniol, 8-oxogeranial, 7-deoxyloganetic acid, 7-deoxyloganic acid and loganic acid, and finally under secologanin synthase *SLS* to generate secologanate [49]. In this study, the levels of the mevalonic acid content along with expression of the key genes in MVA pathway, as well as the genes expression in MEP pathway, were presented lower in LL group compared to ML and HL groups, suggesting that the metabolic flow of iridoids in *G. macrophylla* was prevented under low light. As a result, the content of loganic acid was significantly decreased, as well as the genes expression of *GmGES*, *GmG10H*, *Gm8-HGO*, *GmIS*, *Gm7-DLH* and *GmSLS*. However, the contents of gentiopicroside and sweroside were presented higher in both LL and HL groups compared to ML group. It is difficult to explain that the reason for the biosynthesis pathway from secologanate to gentiopicroside has not been elucidated so far [11]. Considering that plants had regulatory mechanisms to enhance some pivotal metabolites for survival and adaptation in response to changes in the environment [34, 50], we speculated that gentiopicroside and sweroside might be the vital substances for the growth and development of *G. macrophylla*, thus, the metabolic flow from secologanate to gentiopicroside was abnormally activated to maintain the plant survival at low light intensity.

The third consequence is that numerous physiological alterations such as changes in phytohormone levels, plant immune responses, and signal transduction pathways, were induced in response to light stress in plants [33, 51]. As reported, the concentration of abscisic acid (ABA) significantly increased under prolonged dark treatments of light-grown plants of both *Lemna gibba* and *Arabidopsis thaliana* [52]. In addition, exogenous ABA was found to accelerate the degradation of Chls and the accumulation of MDA, leading leaves to turn yellow easily [53]. Our results were similar to these that the expression of genes involved in the biosynthesis of ABA was upregulated by continuous exposure to low light (Additional file 5: Fig. S1 and Additional file 6: Fig. S2, Additional file 9: Table S5), then led to *G. macrophylla* leaves turn yellow and withered. We speculated that ABA played a vital role in the response to light stress in *G. macrophylla*. In addition, *trans*-zeatin, another major and ubiquitous cytokinin in higher plants [54], can be regulated exposure to external stimulis such as low light intensity and cold acclimation [55, 56]. Due to an increase expression at low light intensity, we speculated that *trans*-zeatin was involved in response to light stress in *G. macrophylla*. Moreover, hormones have been reported to respond to light stress via MAPK signaling cascade [57]. The genes related to pathogen infection, hydrogen peroxide signaling, and the ethylene, jasmonic acid, and ABA hormone signaling pathways, along with the expression of genes related to wounding-related enzymes, were significantly altered after treatment with different light intensities (Additional file 14: Fig. S7 and Additional file 15: Fig. S8, Additional file 16: Table S8), suggesting that a series of MAPK signaling transductions were triggered in *G. macrophylla* leaves exposed to low or high light. In brief, a complex and extensive regulatory network would be preformed to restore the internal balance when plants perceive various different light intensities.

The last consequence, TFs as essential regulators of the key enzyme genes in the secondary metabolite biosynthetic pathway, can be regulated by light [15, 23, 58]. As reported, *CYP450s*, *MYB*, and *AP2/ERF* genes might be involved in paclitaxel biosynthesis and play an important role in excessive light in *Taxus chinensis* [39]. Co-expressed analysis of TFs in response to light intensity and genes involved in flavonoid biosynthesis revealed that 69 TFs (WRKY, NAC, MYB, bHLH, etc.) were co-expressed with *PAL*, *4CL*, *C4H*, *CHS*, and *CHI* in *Lonicera japonica* [26]. *AaHY5*, as a key regulator of light-induced artemisinin biosynthesis, could control artemisinin production in response to the alteration of light intensity [59]. In our research, the expression of TFs and genes involved in iridoid biosynthesis was also regulated by light intensity, *GmbHLH20* and *GmMYB5* were shown to regulate the biosynthesis of loganic acid in *G. macrophylla* and might affect the expression of genes involved in iridoid biosynthesis. In addition, TFs might form complexes to regulate diverse metabolic and developmental processes in plants [60]. As a negative correlation was presented, the regulatory mechanism of *GmbHLH20* and *GmMYB5* was inferred to be independent.

## Conclusion

**Fig. 12:**
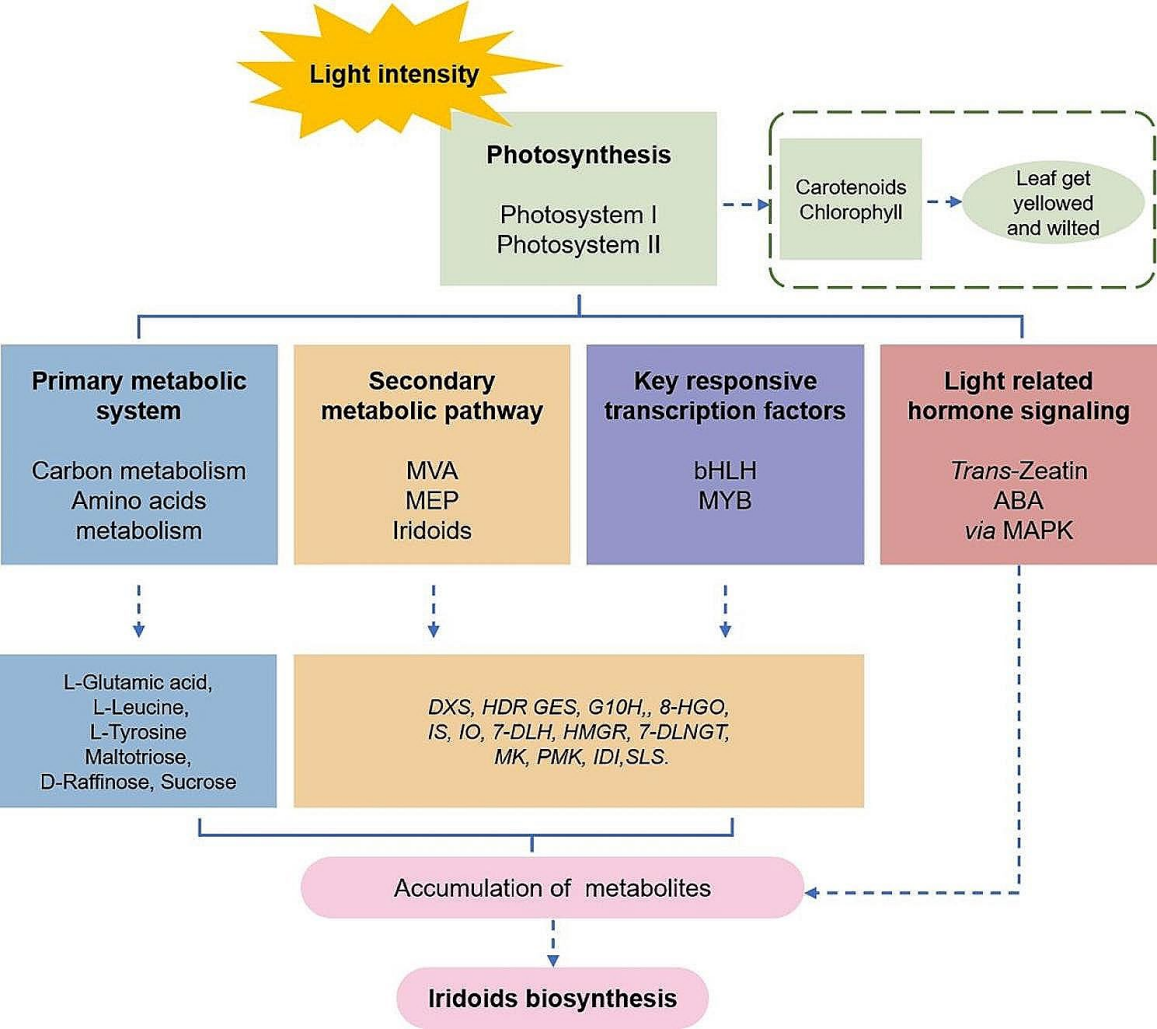
A schematic overview: working model of light regulation on iridoid biosynthesis in *G. macrophylla*

This study represented the first comprehensive investigation of the mechanism of iridoid biosynthesis in *G. macrophylla* under different light intensities by transcriptome and metabolome. A model was proposed to explain the underlying molecular mechanism (Fig. 12). Initially, with the decrease of light intensity, the rate of photosynthesis was primarily decreased, the levels of Chls and carotenoid were inhibited as well as their biosynthesis genes, resulting in the seedlings gradually turning yellow and withered. Subsequently, the alteration of photosynthesis regulated the primary metabolic system, the secondary metabolic pathway, the key responsive TFs and light related hormone signaling, and finally affected the accumulation of iridoids. It was a complex and extensive regulatory network, in which two TFs of *GmMYB5* and *GmbHLH20*, were determined to play crucial roles in the accumulation of iridoid metabolites in response to light stress in *G. macrophylla*. Our research reveals the interplay between photosynthesis and iridoid biosynthesis in *G. macrophylla*, and helps us understand the potential mechanism of ecological factors modulating metabolites accumulation.

### Electronic supplementary material

Below is the link to the electronic supplementary materials.

Supplementary Material 1

Supplementary Material 2

Supplementary Material 3

Supplementary Material 4

Supplementary Material 5

Supplementary Material 6

Supplementary Material 7

Supplementary Material 8

Supplementary Material 9

Supplementary Material 10

Supplementary Material 11

Supplementary Material 12

Supplementary Material 13

Supplementary Material 14

Supplementary Material 15

Supplementary Material 16

## Data Availability

The data presented in the study are deposited in the NCBI repository with the link https://www.ncbi.nlm.nih.gov/. The accession number is PRJNA1043239. All data included in this study are available upon request by contact with the corresponding author.

## Abbreviations

LED: Light-emitting diodes
DEGs: Differentially expressed genes
DAMs: Differentially accumulated metabolites
Chl: Chlorophyll
ML: Medium light
HL: High light
LL: Low light
TF: Transcription factor
MEP: Methyl-erythrose 4-phosphate pathway
MVA: Mevalonate pathway
ABA: Abscisic acid
MAPK: Mitogen activated protein kinase
GO: Gene ontology
KEGG: Kyoto encyclopedia of genes and genomes
FDR: False discovery rate
NCBI: National center for biotechnology information
Nr: Non-redundant
PPFD: The photosynthetic photon flux density
RT-qPCR: Quantitative reverse transcription PCR
