# Supplementary figures and images for: Transcriptome and metabolome analysis reveals mechanism of light intensity modulating iridoid biosynthesis in Gentiana macrophylla Pall
Source: BMC Plant Biol. 2024 Jun 11;24:526. doi: 10.1186/s12870-024-05217-y (PMC11165902; doi:10.1186/s12870-024-05217-y)

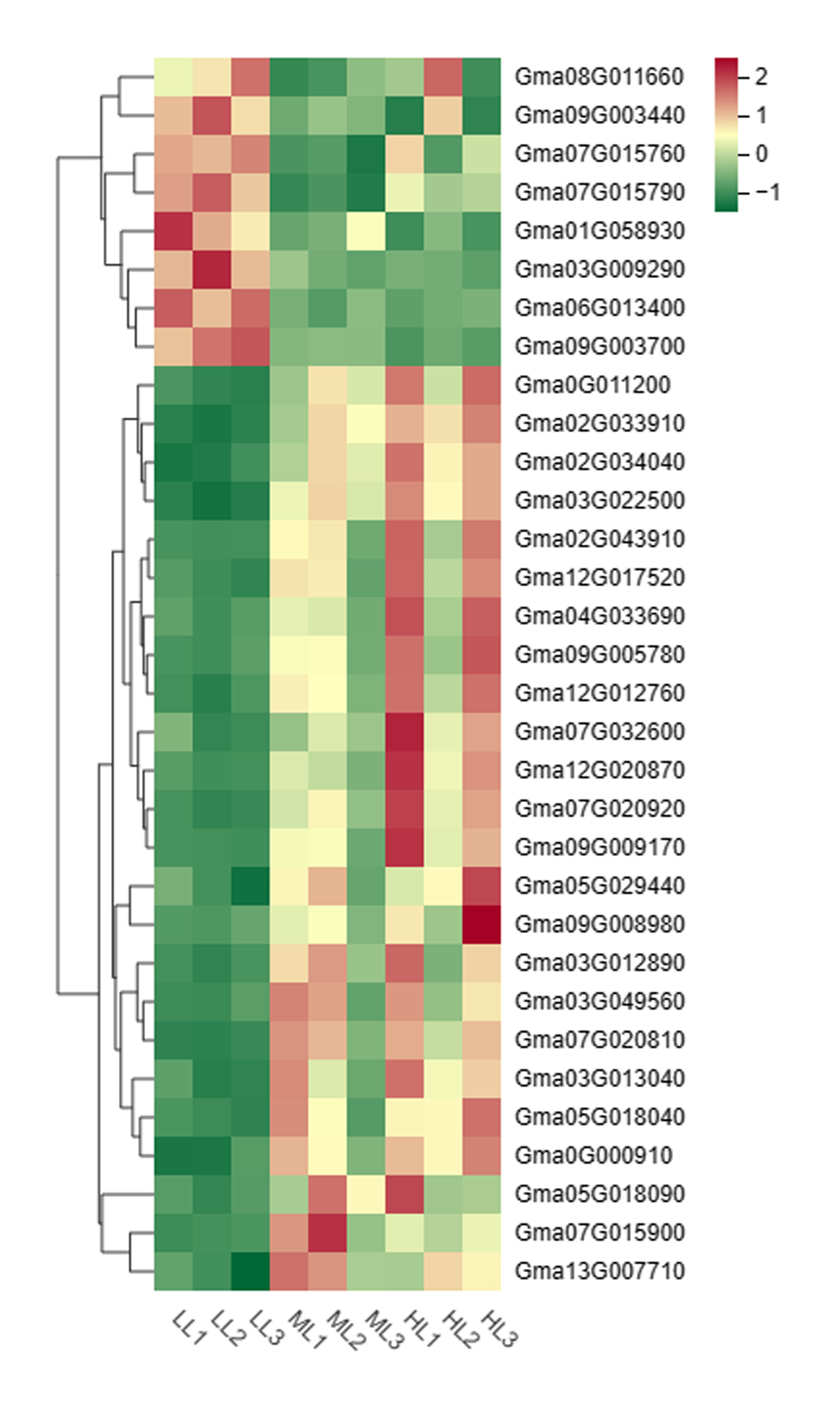

Supplement: Supplementary file 6 — Supplementary Material 6 [file 12870_2024_5217_MOESM6_ESM.tif]

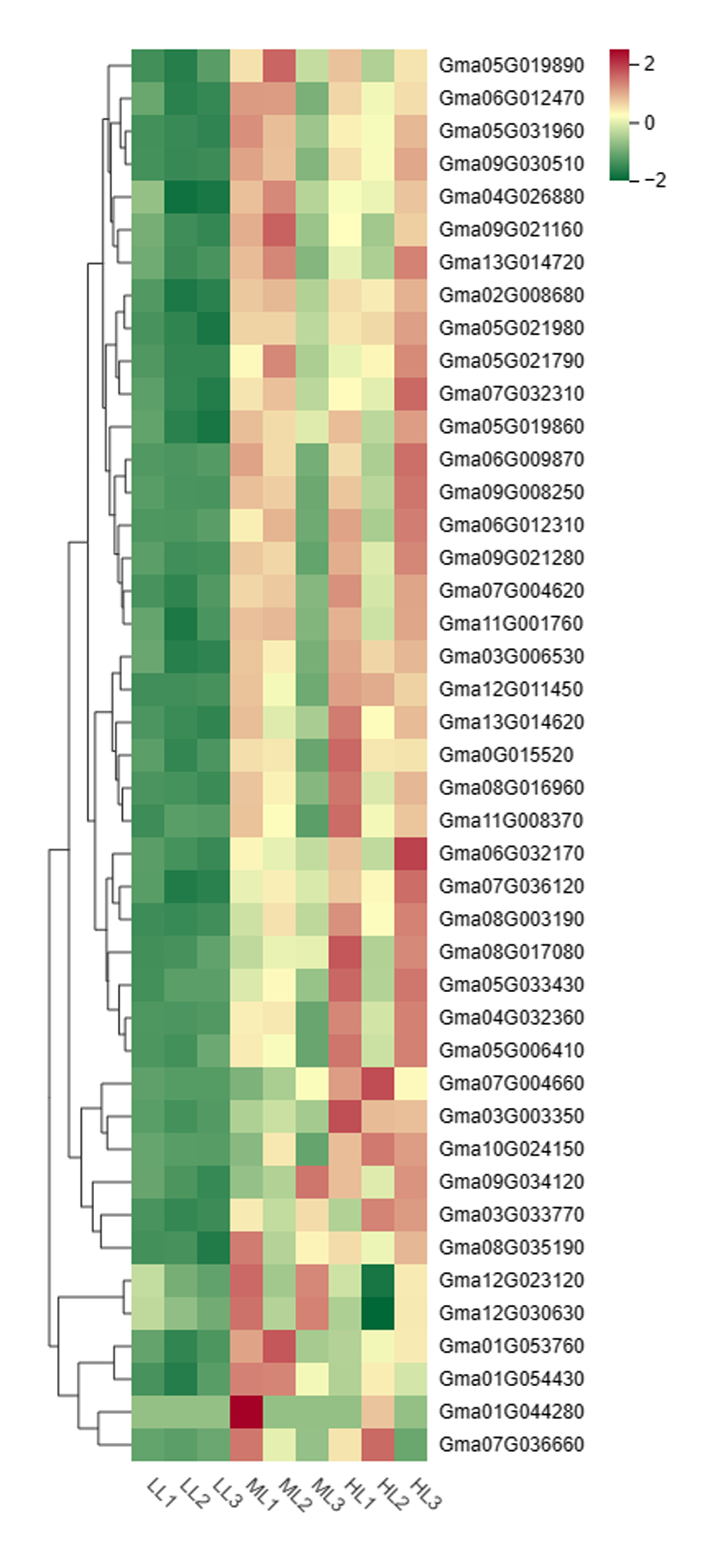

Supplement: Supplementary file 8 — Supplementary Material 8 [file 12870_2024_5217_MOESM8_ESM.tif]

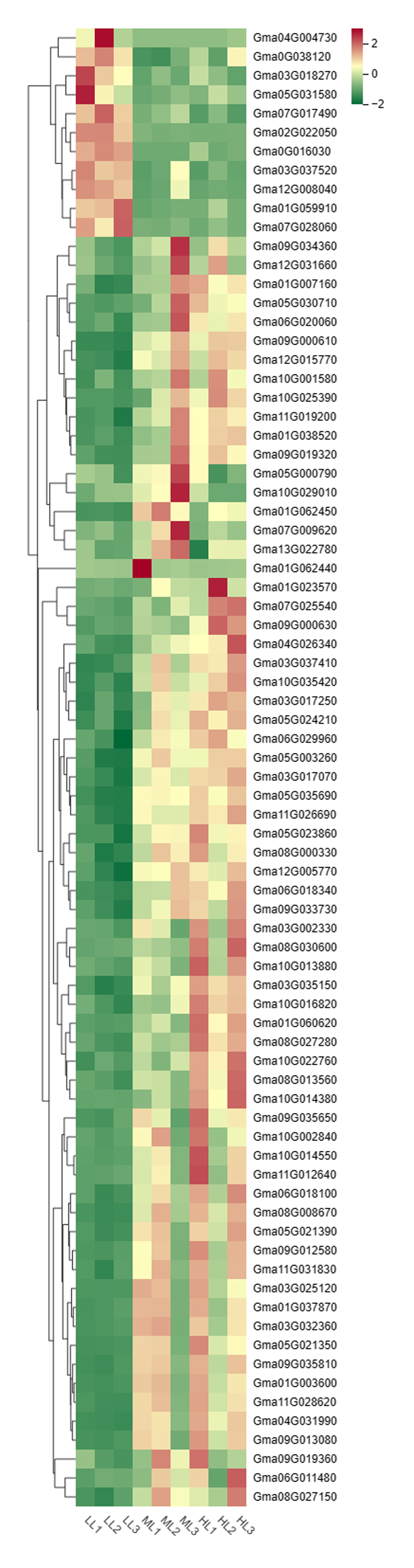

Supplement: Supplementary file 12 — Supplementary Material 12 [file 12870_2024_5217_MOESM12_ESM.tif]

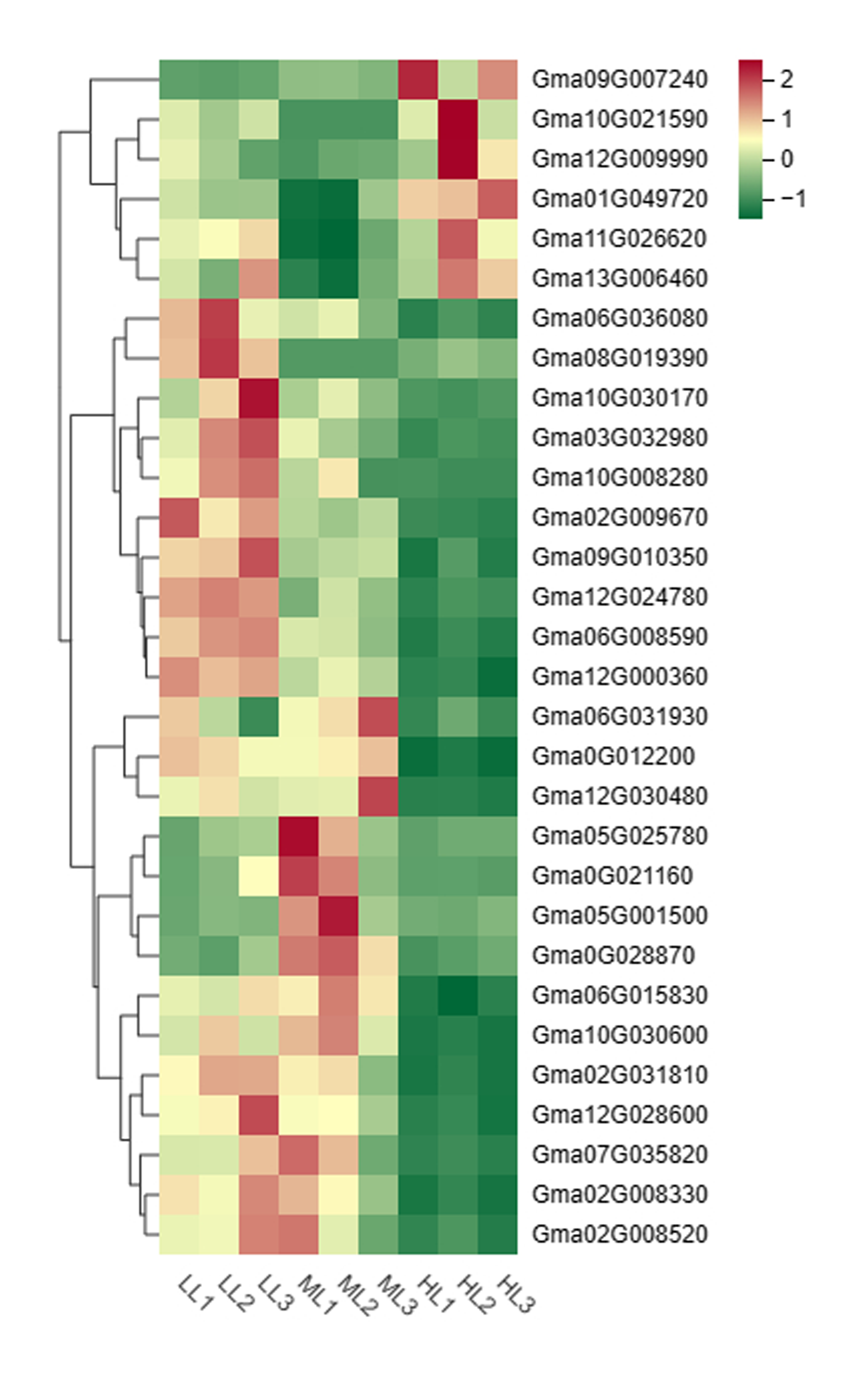

Supplement: Supplementary file 15 — Supplementary Material 15 [file 12870_2024_5217_MOESM15_ESM.tif]
